# Proinflammatory Chemokine Levels in Cerebrospinal Fluid of Patients with Neuroinvasive Flavivirus Infections

**DOI:** 10.3390/microorganisms12040657

**Published:** 2024-03-26

**Authors:** Snjezana Zidovec-Lepej, Kristian Bodulić, Maja Bogdanic, Lana Gorenec, Vladimir Savic, Ivana Grgic, Dario Sabadi, Marija Santini, Leona Radmanic Matotek, Jasmina Kucinar, Ljubo Barbic, Ljiljana Zmak, Thomas Ferenc, Vladimir Stevanovic, Ljiljana Antolasic, Ljiljana Milasincic, Zeljka Hruskar, Mateja Vujica Ferenc, Tatjana Vilibic-Cavlek

**Affiliations:** 1 Department of Immunological and Molecular Diagnostics, University Hospital for Infectious Diseases “Dr. Fran Mihaljevic”, 10000 Zagreb, Croatia; szidovec@gmail.com (S.Z.-L.); lgorenec@gmail.com (L.G.); ivanahobby@gmail.com (I.G.); leona.radmanic@gmail.com (L.R.M.); 2 Research Department, University Hospital for Infectious Diseases “Dr. Fran Mihaljevic”, 10000 Zagreb, Croatia; kristian.bodulic@gmail.com; 3 Department of Virology, Croatian Institute of Public Health, 10000 Zagreb, Croatia; maja.bogdanic@hzjz.hr (M.B.); ljiljana.antolasic@hzjz.hr (L.A.); ljiljana.milasincic@hzjz.hr (L.M.); zeljka.hruskar@hzjz.hr (Z.H.); 4 Poultry Center, Croatian Veterinary Institute, 10000 Zagreb, Croatia; v_savic@veinst.hr; 5 Department of Infectious Diseases, Clinical Hospital Center Osijek, 31000 Osijek, Croatia; dariocroatia@gmail.com; 6 Faculty of Medicine, Josip Juraj Strossmayer University of Osijek, 31000 Osijek, Croatia; 7 Department for Infections in Immunocompromised Patients, University Hospital for Infectious Diseases “Dr. Fran Mihaljevic”, 10000 Zagreb, Croatia; marijasantini.ms@gmail.com; 8 School of Medicine, University of Zagreb, 10000 Zagreb, Croatia; ljiljana.zmak@hzjz.hr; 9 Department of Serology and Immunology, Istria County Institute of Public Health, 52100 Pula, Croatia; jasmina.kucinar@zzjziz.hr; 10 Department of Microbiology and Infectious Diseases with Clinic, Faculty of Veterinary Medicine, University of Zagreb, 10000 Zagreb, Croatia; ljubo.barbic@vef.hr (L.B.); vladostevanovic@gmail.com (V.S.); 11 Department of Microbiology, Croatian Institute of Public Health, 10000 Zagreb, Croatia; 12 Department of Diagnostic and Interventional Radiology, Merkur University Hospital, 10000 Zagreb, Croatia; 13 Department of Obstetrics and Gynecology, University Hospital Centre Zagreb, 10000 Zagreb, Croatia; matejavujica1@gmail.com

**Keywords:** chemokines, neuroinvasive diseases, cerebrospinal fluid, tick-borne encephalitis virus, West Nile virus

## Abstract

Tick-borne encephalitis virus (TBEV) and West Nile virus (WNV) are the most important neuroinvasive arboviruses detected in Europe. In this study, we analyzed cerebrospinal fluid (CSF) concentrations of 12 proinflammatory chemokines (CCL2, CCL3, CCL4, CCL11, CCL17, CCL20, CXCL1, CXCL5, CXCL8, CXCL9, CXCL10, and CXCL11) in 77 patients with neuroinvasive diseases (NIDs). Flavivirus infection was confirmed in 62 patients (TBEV and WNV in 31 patients each), while in 15 patients the etiology of NID was not determined (NDE). Similar patterns of high-level expression of chemokines regulating monocyte/macrophage responses (CCL2), neutrophil recruitment (CXCL1 and CXCL8), and interferon-inducible chemoattractants for leukocytes (CXCL10 and CXCL11) have been observed in WNV and TBEV groups. None of the tested chemokines significantly differed between patients with TBEV or WNV. Concentrations of CCL17, CCL20, CXCL5, CXCL10, and CXCL11 were significantly lower in both WNV and TBEV groups compared to NID NDE patients. The logistic regression model showed that CSF concentrations of CXCL11, CXCL5, and CXCL10 could potentially be used for the classification of patients into the WNV or TBEV group versus groups with other NIDs. This study identified, for the first time, similar patterns of CSF chemokine expression in WNV and TBEV infections, suggesting common immunopathogenic mechanisms in neuroinvasive flavivirus infections that should be further evaluated.

## 1. Introduction

Flaviviruses, such as tick-borne encephalitis virus (TBEV) and West Nile virus (WNV), are some of the most common neuroinvasive arboviruses and represent an important public health problem in many European countries. From 2012 to 2020, 19 European Union/European Economic Area (EU/EEA) countries reported 29,974 tick-borne encephalitis (TBE) cases, of which 98.6% of infections were autochthonous. In addition to the increased number of cases, a northwest spread in continental Europe was observed [1]. In the past two decades, WNV infections have continuously been recorded in the EU/EEA. In 2018, intense WNV circulation was observed, when the number of reported autochthonous infections (2083 cases) exceeded the total number from the previous seven seasons (1832 recorded infections) [2]. In addition, in 2022, the EU/EEA reported the highest number of locally acquired WNV cases since the peak epidemic year of 2018 [3]. Since both viruses can result in long-term neurological sequelae and even fatal outcomes, TBEV and WNV represent emerging health threats [4].

A high proportion of TBE cases are asymptomatic (>70%). The course of the infection caused by the TBEV European subtype (TBEV-Eu) is usually biphasic, while the TBEV Far East (TBEV-FE) and Siberian (TBEV-Si) subtypes cause monophasic disease. About 50% of patients with a neuroinvasive form of TBE develop meningitis, 40% develop encephalitis, and 5–10% develop myelitis [5]. The mortality is highest for the TBEV-FE (~20%), compared to 1–2% for the TBEV-Eu and 1–3% for the TBEV-Si [6]. Although the majority of human WNV infections are asymptomatic or present as a mild febrile disease (WNV fever), some patients, especially elderly and immunocompromised, may develop neuroinvasive disease (WNND). The main clinical presentations of WNND include meningitis, meningoencephalitis, and meningoencephalomyelitis. The mortality rates in patients with WNND may be up to 10% [7,8].

In Croatia, flavivirus infections are regularly reported in the continental counties. TBE is considered endemic in northwestern and eastern regions with 84 autochthonous cases reported from 2017 to 2023 [9]. WNV infections occur sporadically or as smaller (2012, 2022, 2023) and larger (2013, 2018) outbreaks. WNV patients were detected in almost all continental Croatian counties [10,11]. While epidemiological and clinical characteristics are studied continuously, only two studies analyzed the cytokine response in different clinical samples of patients with TBEV and WNV infections [12,13]. However, data on the chemokine antiviral response are lacking.

Chemokines are a large family of chemotactic cytokines that are classified into four groups (CC, CX3C, CXC, and XC), based on the spacing between conserved cysteine motifs. These immunological mediators exert their biological activity via G protein-coupled heptahelical chemokine receptors. Although the most studied biological function of chemokines is their impact on cellular migration (particularly leukocytes), chemokines also regulate the biology of various cell types, contribute to the development and homeostasis of the immune system, and are involved in the immunopathogenesis of many infectious and non-infectious diseases [14].

Neuroinvasive viral infections are characterized by the intrathecal synthesis of various proinflammatory chemokines, cytokines, and growth factors that mediate antiviral immunity but, on the other hand, may contribute to the pathogenesis of disease. Acute WNV infection activates several pathogen recognition receptors that recognize viral RNA, including Toll-like receptors (TLR)-3 and -7, NOD-like receptors containing pyrin domain (NLRP), and RIG-I-like receptors (RLR), leading to the synthesis of class I and III interferons, proinflammatory cytokines, and chemokines [15]. The WNV invasion into the central nervous system (CNS) and subsequent infection of neurons, microglia, and astrocytes, further contribute to the synthesis of chemokines and cytokines that mediate antiviral immunity (both innate and subsequently specific) but also enhance disease pathology [16]. Data from in vitro studies and animal models suggest an important contribution of several chemokines (CCL2, CCL7, CXCL10, and CCR5) in WNND immunopathology, including, but not limited to, virus-induced monocytosis and recruitment of monocytes, neutrophils, as well as virus-specific CD8+ T-cells, into the CNS [15].

Recently, we described the pattern of intrathecal cytokine expression in patients with WNV fever and WNND presenting with meningitis or meningoencephalitis that is characterized by increased concentrations of proinflammatory cytokine IL-6 and the absence of IL-2, IL-4, TNF-α, and Th17 cytokines in the cerebrospinal fluid (CSF) of WNV-infected patients [8,13]. However, the literature data on the expression of chemokines in human WNV infection are limited to studies involving blood donors (asymptomatic or preceding the occurrence of symptoms) and patients with the post-infectious syndrome after WNV infection with no available data on WNND [17,18,19,20].

Contrary to this, a large number of studies analyzed the expression of chemokines, cytokines, and growth factors in the CSF, serum, and urine of TBE patients with a particular interest in the intrathecal expression of these mediators in the meningoencephalitic stage of disease and the serum/CSF chemotactic gradient. The goal of these studies was to evaluate the potential role of chemokines as biomarkers of TBE severity or mortality [21,22,23,24,25,26,27,28,29,30,31,32,33]. In a recent study, we compared cytokine levels in the serum, CSF, and urine of TBE patients showing major differences in cytokines regulating early innate immune response cytokines, Th1, Th2, Th9, Th22, and Th17, and anti-inflammatory cytokines [12].

The literature data on the comparison of the complex patterns of proinflammatory intrathecal chemokine synthesis in patients with severe flavivirus neuroinvasive diseases such as WNND and TBE are currently not available. This study aimed to compare the expression patterns of 12 proinflammatory chemokines in the CSF of WNND and TBE patients, as well as in patients presenting with neuroinvasive disease of undetermined etiology, and find possible similarities in the chemokine responses to WNV and TBEV in the CNS. We analyzed a comprehensive group of proinflammatory chemokines, including chemokines involved in monocyte/macrophage responses (CCL2, CCL3, and CCL4), lymphocyte chemoattractants (CCL17 and CCL20), regulators of neutrophil recruitment (CXCL1, CXCL5, and CXCL8), interferon-inducible chemoattractants for leukocytes (CXCL9, CXCL10, and CXCL11), and CCL11 that regulates the biology of eosinophils.

## 2. Materials and Methods

### 2.1. Patients

This study included a total of 77 patients with neuroinvasive diseases tested in the period from 2017 to 2023. In all patients, the CSF examination revealed pleocytosis with mononuclear predominance, elevated protein level, and normal glucose level, suggesting a viral etiology of neuroinvasive disease. CSF samples were tested (PCR/RT-PCR) for most common neurotropic viral infections, including herpes simplex viruses (HSV-1/2), varicella-zoster virus, enteroviruses, and neuroinvasive arboviruses, that were detected in Croatia in previous seasons: TBEV, WNV, Usutu virus (USUV), and Toscana virus (TOSV). In addition, serum and CSF samples were tested for TBEV, WNV, USUV, and TOSV IgM and IgG antibodies (enzyme-linked immunosorbent assay; ELISA, indirect immunofluorescence assay; IFA, virus neutralization test; VNT). TBEV and WNV were detected in 31 patients each, while in 15 patients, the etiology remained unknown.

### 2.2. Methods

#### 2.2.1. Flavivirus Detection

The flavivirus diagnosis was confirmed according to the European Centre for Disease Control and Prevention (ECDC) criteria: (a) detection of specific IgM and IgG in serum confirmed by a virus neutralization test (VNT), (b) detection of IgM antibodies in the CSF, (c) detection of viral RNA in the CSF or urine [34].

Viral RNA was detected using a real-time reverse-transcriptase polymerase chain reaction (RT-PCR) assay specific for TBEV (Schwaiger and Casinotti, 2003) [35] and WNV (Tang et al., 2006) [36]. Initial serological screening was performed using commercial ELISA (Euroimmun, Lübeck, Germany). The results were expressed in the ratio (IgM) < 0.8 negative, 0.8–1.1 borderline, >1.1 positive, and RU/mL (IgG): <16 negative, 16–22 borderline, >22 positive. IgM/IgG positive samples were further tested for IgG avidity (Euroimmun, Lübeck, Germany) [37,38]. The IgG avidity index (AI) was calculated and interpreted as follows: <40% low AI (acute/recent infection); 40–60% borderline AI; >60% high AI (previous infection). Samples with cross-reactive flavivirus antibodies were confirmed using a VNT in cell culture [39].

#### 2.2.2. Chemokine Determination

Chemokine determination was performed using a LEGENDplex™ Multi-Analyte Flow Assay Kit (LEGENDplex Human Proinflammatory Chemokine panel, BioLegend, San Diego, CA, USA) that allows simultaneous quantification of 12 human chemokines: MCP-1 (CCL2), MIP-1α (CCL3), MIP-1β (CCL4), eotaxin (CCL11), TARC (CCL17), MIP-3α (CCL20), GROα (CXCL1), ENA-78 (CXCL5), IL-8 (CXCL8), MIG (CXCL9), IP-10 (CXCL10), and I-TAC (CXCL11). The flow cytometry was performed on the FACS Canto II instrument (Beckton Dickinson, Franklin Lakes, NJ, USA). Minimum detectable assay concentrations for the selected cytokines (pg/mL) were CXCL8 3.4 ± 1.7, CXCL10 1.2 ± 0.9, CCL11 2.5 ± 4.8, CCL17 1.0 ± 0.9, CCL2 1.4 ± 1.4, CCL3 3.8 ± 2.6, CXCL9 0.5 ± 0.2, CXCL5 0.6 ± 0.6, CCL20 0.4 ± 0.4, CXCL1 0.5 ± 0.4, CXCL11 0.4 ± 0.3, and CCL3 0.3 ± 0.3. The CSF samples were stored at −80 °C in aliquots to avoid freeze-and-thaw cycles.

#### 2.2.3. Statistical Analysis

Distributions of numerical variables were tested for normality by graphical assessment. Two-group comparisons between numerical variables were performed using the Mann–Whitney U test. Multiple group comparisons of numerical variables were performed using Kruskal–Wallis and Dunn’s post hoc test. Correlation between numerical variables was evaluated using Spearman’s correlation coefficient and the correlation test. Association between categorical variables was assessed using the Fisher’s exact test or chi-square test, as appropriate. *p-values* were corrected for multiple comparisons using the Benjamini–Hochberg procedure. Patients with confirmed and undetermined etiology were classified with binary logistic regression models. Chemokines best at separating the groups were identified by the best subset selection algorithm, using the models’ maximal Bayesian information criterium (BIC) as the selection criterion. All chemokine levels were logarithmically scaled (base 2) to ensure residual heteroskedasticity. The receiver operating characteristic (ROC) curve was generated using sensitivity and specificity values obtained by five-fold cross-validation. Confidence intervals for the area under the curve (AUC) were calculated using DeLong’s method. All statistical tests were two-tailed with a significance level of 95%. Data analysis was performed using R (version 4.2.3.) with packages ggplot2 (version 2.3.3.), ggpubr (version 0.4.0.), bestglm (version 0.37.3.), and pROC (version 1.18.5.) [40].

## 3. Results

### 3.1. Patient Characteristics

Demographic and clinical characteristics of patients with confirmed flavivirus infections are presented in Table 1. A total of 38 (61%) patients were male, with a median age of 58 years (range 12–88 years). There were no significant differences in the sex distribution of the patients infected with TBEV and WNV (68% vs. 55% males, *p* = 0.435). However, TBEV-infected patients were significantly younger than patients infected with WNV (median ages: 46 vs. 63 years, *p* = 0.008). The most common clinical presentations were meningitis (50%) and meningoencephalitis (37%). There was no significant difference in the clinical presentations among patients infected with TBEV and WNV (*p* = 0.545). Three (10%) patients infected with WNV died. In 15 individuals included in this study (40% males, median age 63 years), the etiology of neuroinvasive disease remained unknown. There was no significant difference between the sex distribution in the patients with confirmed flavivirus infection and patients with undetermined etiology (*p* = 0.156). Similarly, there was no significant age difference between the patients in both groups (*p* = 0.354).

### 3.2. Chemokine Levels Comparison According to Demographic Characteristics and Clinical Diagnosis

**Figure 1:**
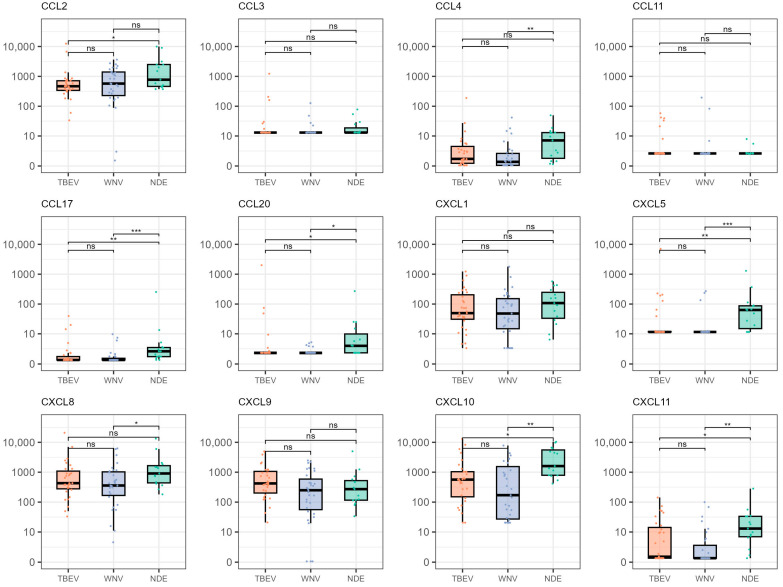
Distribution of chemokine levels in patients infected with TBEV, patients infected with WNV, and patients with NDE. The boxes show the median and interquartile range of the distribution, whereas the whiskers extend to the minimum and maximum nonoutlier values of the distribution. Chemokine levels are given in pg/mL. The Y-axis is logarithmically scaled. ***: *p* < 0.001, **: *p* < 0.01, *: *p* < 0.05, ns: *p* > 0.05 (Dunn’s post hoc test, *p*-values adjusted with the Benjamini–Hochberg method). TBEV = tick-borne encephalitis virus, WNV = West Nile virus, NDE = not determined etiology.

The analyzed chemokines did not show a significant correlation with age (−0.20 < r < 0.20, *p* > 0.05) or a significant difference in male and female patients (*p* > 0.05). The comparison of chemokine levels in patients infected with TBEV, patients infected with WNV, and patients with not determined etiology (NDE) of neuroinvasive disease is presented in Figure 1. None of the analyzed chemokines significantly differed in patients infected with TBEV and WNV (*p* > 0.05). Notably, this was the case even before adjusting the *p*-values for multiple comparisons. However, TBEV- and WNV-infected patients generally showed lower chemokine levels than patients with NDE. When compared to this group, patients infected with TBEV displayed significantly lower levels of CCL2 (medians 466.81 and 773.80 pg/mL, *p* = 0.033), CCL17 (medians 0.63 and 2.24 pg/mL, *p* = 0.005), CCL20 (medians 1.90 and 3.74 pg/mL, *p* = 0.020), CXCL5 (medians 11.56 and 63.33 pg/mL, *p* = 0.007), CXCL10 (medians 563.92 and 1601.30 pg/mL, *p* = 0.014), and CXCL11 (medians 0.86 vs. 13.08 pg/mL, *p* = 0.021). Similarly, patients infected with WNV exhibited significantly lower levels of CCL4 (medians 0.65 and 7.04 pg/mL, *p* = 0.007), CCL17 (medians 0.63 and 2.24 pg/mL, *p* < 0.001), CCL20 (medians 1.90 and 3.74 pg/mL, *p* = 0.013), CXCL5 (medians 11.56 and 63.33 pg/mL, *p* < 0.001), CXCL8 (medians 358.18 and 905.50 pg/mL, *p* = 0.033), CXCL10 (medians 170.44 and 1601.30 pg/mL, *p* = 0.002), and CXCL11 (medians 0.62 and 13.08 pg/mL, *p* = 0.001) than patients with NDE. When comparing the analyzed chemokine levels among the patients with meningitis and meningoencephalitis, none of the analyzed chemokines showed significant differences (*p* > 0.05). This was also true when stratifying the patient groups by detected virus. When considering the patient outcome, we did not record a significant difference in surviving and deceased patients in any of the analyzed chemokine levels (*p* > 0.05).

### 3.3. Correlation Analysis of the Selected Chemokines

**Figure 2:**
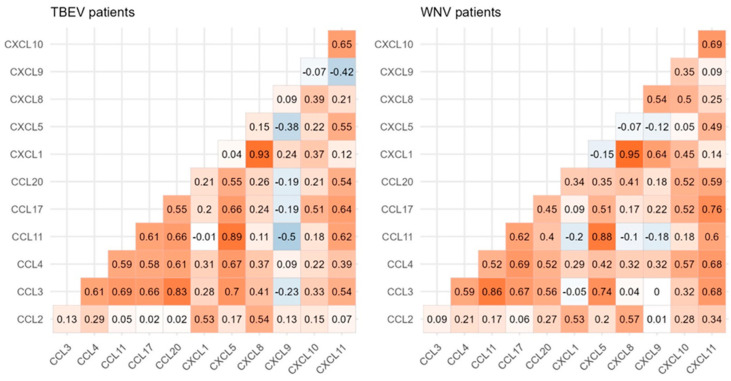
Correlation matrix of the analyzed chemokines in patients infected with TBEV and WNV. The given values correspond to the Spearman correlation coefficient between the corresponding chemokines.

Following chemokine level comparisons, the potential pairwise correlations between the selected chemokines were also evaluated (Figure 2). Chemokine correlations were relatively similar between patients infected with TBEV and WNV. These similarities include a strong positive correlation between CXCL1 and CXCL8 (TBEV: r = 0.93, *p* < 0.001, WNV: r = 0.95, *p* < 0.001), as well as a strong positive correlation between CCL3 and several chemokines: CCL11 (TBEV: r = 0.69, *p* < 0.001, WNV: r = 0.86, *p* < 0.001), CCL17 (TBEV: r = 0.66, *p* < 0.001, WNV: r = 0.67, *p* < 0.001), and CXCL5 (TBEV: r = 0.70, *p* < 0.001, WNV: r = 0.74, *p* < 0.001). We also found a strong positive correlation in both patient groups between CCL11 and three chemokines: CCL17 (TBEV: r = 0.61, *p* < 0.001, WNV: r = 0.62, *p* < 0.001), CXCL5 (TBEV: r = 0.89, *p* < 0.001, WNV: r = 0.88, *p* < 0.001), and CXCL11 (TBEV: r = 0.62, *p* < 0.001, WNV: r = 0.60, *p* < 0.001). Patients infected with TBEV exhibited a moderate negative correlation between CCL11 and CXCL9 (r = −0.50, *p* < 0.001) and between CXCL5 and CXCL9 (r = −0.38, *p* = 0.009), where no such correlations were found in patients infected with WNV (*p* > 0.05). Another notable difference between the patient groups was a strong positive correlation between CCL4 and CXCL11 in WNV patients (r = 0.68, *p* < 0.001) and a moderate positive correlation between CCL4 and CXCL10 in WNV patients (r = 0.57, *p* < 0.001), both of which were not recorded in TBEV patients (*p* > 0.05).

### 3.4. Multivariate Analysis of the Selected Chemokines

Finally, we used binary logistic regression to identify the chemokines best distinguishing the analyzed patients in a multivariate context (Table 2). Considering that patients infected with TBEV and WNV did not show significant differences in any of the analyzed chemokines, logistic regression was utilized to classify two groups: patients infected with TBEV or WNV and patients with NDE. The model best separating the groups included three chemokines: CCL11, CXCL5, and CXCL10. Doubling the levels of CCL11 would increase the odds of a participant belonging to the patient group by 14.5% (95% CI 6.9–21.4%, *p* < 0.001). Doubling the levels of CXCL5 would decrease the odds of a participant being in the patient group by 15.5% (95% CI 8.0–23.5%, *p* < 0.001). Similarly, doubling the levels of CXCL10 would decrease the odds of a participant belonging to the patient group by 4.4% (95% CI 1.4–7.4%, *p* = 0.004). This model achieved an accuracy of 72.7% (sensitivity: 74.2%, specificity: 66.7%) and an AUC of 0.84 (95% CI 0.70–0.93) when classifying the flavivirus-infected group and the group with other neuroinvasive diseases. Adding other chemokines as predictors did not significantly improve the model accuracy.

## 4. Discussion

The results of this study have shown, for the first time, concordant patterns of CSF chemokines in WNND and TBE that are characterized by the high-level expression of chemokines involved in monocyte/macrophage responses (CCL2), regulators of neutrophil recruitment (CXCL1 and CXCL8), and interferon-inducible chemoattractants for leukocytes (CXCL10 and CXCL11). Concentrations of CCL17, CCL20, CXCL5, CXCL10, and CXCL11 were significantly lower in both WNND and TBE groups compared to patients with neuroinvasive disease of NDE. By using a multivariate analysis, we have also shown that CSF concentrations of CCL11, CXCL5, and CXCL10 can potentially be used for patient classification in the WNND or TBE group.

In this study, we analyzed three interferon-inducible chemokines, CXCL9 (monokine induced by interferon-γ), CXCL10 (interferon-γ-inducible protein 10), and CXCL11 (interferon-inducible T-cell alpha chemoattractant) that exert their biological effect by CXCR3-mediated signaling. These chemokines are synthesized by a variety of cells including astrocytes and play an important role in the differentiation and recruitment of leukocytes to sites of inflammation [41].

The importance of the CXCL10 and CXCL11 concentration gradient between CSF and serum as a biological mechanism that is responsible for the recruitment of CXCR3-expressing T-cells as principal mediators of local antiviral response into the CNS of TBE patients was first proposed by our group in 2007 [21]. Several subsequent studies confirmed these findings, including a study by Bogovič et al. (2022) [29] showing an elevated expression of CXCL11 and CXCL13 (a chemokine regulating the biology of B-cells) in the serum of TBE patients along with a distinct cytokine/chemokine expression pattern in the CSF that included CXCL10 and CCL19. In another study, Bogovič et al. (2021) [42] found higher CSF concentrations of CXCL9 in 35 TBE patients with a monophasic course compared to 46 patients with a biphasic course of disease.

Contrary to these findings, the literature data on the expression of CXCL9, CXCL10, and CXCL11 in the CSF of WNND patients are not available, and our results provide the first evidence of the similarities in the expression profile of these three chemokines in the CSF of WNND and TBE patients. Despite the lack of data on WNND, two studies in WNV-infected blood donors have shown increased systemic concentrations of CXCL10 and CXCL10 compared to healthy controls [17,18]. By using a decision tree analysis, Fares-Gusmao et al. (2019) [18] have shown that plasma concentrations of CXCL10, as well as IL-1ra, P-selectin, and IL-10, enabled the correct classification of blood donors as non-infected controls versus presymptomatic/asymptomatic donors infected with WNV, dengue virus, or Zika virus. Hoffman et al. (2016) [19] have shown significantly increased CXCL10 concentrations in WNV patients experiencing milder clinical presentation of WNV infection (no symptoms or one symptom) compared with patients experiencing two or more symptoms, suggesting a possible favorable role of sustained proinflammatory cytokine synthesis on the clinical presentation of disease. However, this interpretation is challenged by Garcia et al. (2014) [20] showing significantly higher concentrations of CXCL10 in patients with prolonged post-infection fatigue (longer than six months) following symptomatic WNV infection. These results suggest that acute WNV infection is associated with increased systemic concentrations of CXCL10 and CXCL9 and is in concordance with our observation of high concentrations of these two chemokines in the CSF of WNND and TBE patients. This suggests an important role of the stated chemokines in inflammatory responses to neuroinvasive CNS infections.

Our study identified CXCL10 and CXCL11 as biomarkers for patients’ classification to WNND and TBE groups versus patients with other neuroinvasive diseases. Interestingly, in a study that included 23 patients (TBE and patients with excluded TBE/other inflammatory CNS diseases), Zajkowska et al. (2011) [23] found that serum concentrations of CXCL10, as well as CSF concentrations of CXCL10, CXCL11, and CXCL12, can be used to differentiate the patients between the two groups. Additionally, CSF concentrations of CXCL10 and CXCL11 in the CSF of TBE patients were significantly different before and after treatment, pointing towards their possible use as biomarkers of recovery. In another study, Bogovič et al. (2019) [33] showed higher serum concentrations of Th1 mediators (including CXCL9 and CXCL10) in TBE patients with post-encephalitic syndrome, suggesting that these chemokines could be important as markers of unfavorable clinical outcome. The literature data on the value of CXCL9, CXCL10, and CXCL11 as biomarkers of WNND clinical presentation are not currently available. Therefore, further evaluation of CXCL10 and CXCL11 CSF expression as biomarkers of clinical severity and outcome in larger WNND and TBE patient cohorts is recommended.

Neutrophil infiltration is a common pathogenic finding in the CSF of patients with TBE (particularly in the early stages of disease) and WNDD (more than 50% of neutrophils detectable in 40–50% of patients) [43,44]. Therefore, we analyzed the expression of three glutamic acid+leucine+arginine (ELR+) CXC chemokines that represent neutrophil chemoattractants; CXCL1, CXCL5, and CXCL8. These chemokines use chemokine receptors CXCR1 and/or CXCR2 expressed on polymorphonuclear leukocytes, epithelial, and endothelial cells [45]. Our study has shown high concentrations of CCL1 and CCL8 in the CSF of WNND and TBE patients and identified CXCL5 as a possible biomarker that allows the classification of patients as having WNDD or TBE. Grygorczuk et al. (2018) [25] previously showed that high intrathecal synthesis of CXCL8 and CXCL1 correlated with neutrophil counts and was more pronounced in patients with encephalitis, suggesting an important role of these chemokines as mediators of neutrophil migration into the CSF in TBE. Contrary to these findings, the literature data on the expression of chemokines involved in neutrophil CNS responses in WNND are not currently available. Our results showing a similarity in the patterns of CXCL1, CXCL5, and CXCL8 expression in the CSF of TBE and WNND patients suggest that the three chemokines play an important role in facilitating neutrophil infiltration in patients with neuroinvasive CNS infections.

We also analyzed the expression of chemokines CCL2, CCL3, and CCL4, which are synthesized in response to infection and play an important role in the recruitment of monocytes/macrophages to inflammation sites. Both CCL3 (macrophage inflammatory protein-1α) and CCL4 (macrophage inflammatory protein-1β) bind to the chemokine receptor CCR5 that is expressed on macrophages, dendritic cells, and activated Th1 lymphocytes. Notably, CCL3 also mediates signaling via CXCR1, while CCL2 (monocyte chemoattractant protein-1) binds to the receptor CCR2. Regarding this chemokine group, we observed significantly lower expression of CCL2 in WNND and CCL4 in TBE patients in comparison with patients with neuroinvasive diseases of NDE. These differences can be, in part, explained by overlapping, but not the identical target cells that the stated chemokines attract to sites of inflammation. Furthermore, the differences could also be partially explained by different types of regulatory activity in the differentiation of T-cells with CXCL2 stimulating non-protective Th2 type responses [46]. Grygorczuk et al. (2006) [47] found significantly lower concentrations of CCL3 in the CSF compared with the serum of TBE patients, suggesting the lack of involvement of this chemokine in the pathogenesis of TBE. Importantly, our results represent the first available data on CCL2, CCL3, and CCL4 expression in WNND.

Our analysis of chemokines that predominantly act as chemoattractants for lymphocytes showed significant differences between WNND and TBE in comparison with neuroinvasive diseases of NDE. CCL17 is a chemokine that recruits lymphocytes that express high levels of receptor CCR4, such as Th2 cells and regulatory T-cells, to sites of immune responses [48]. Although the majority of experimental data on CCL17 in human diseases are focused on cancer, elevated concentrations of this chemokine have also been detected in autoimmune and inflammatory processes. CXCL20 (liver activation-regulated chemokine) is a chemokine with a strong chemotactic activity for lymphocytes that binds to the receptor CCR6. Signaling via the CCL20/CCR6 axis has been implicated in the CNS autoimmune pathogenesis as a mechanism for the recruitment of Th17 cells and regulatory T cells to the sites of inflammation. However, recent studies on experimental autoimmune encephalomyelitis involving CCL20-knock-out mice suggest that this signaling deficiency may be compensated by mechanisms of chemokine redundancy [49]. Our results suggest that CCL17 and CXCL20 as mediators of lymphocyte recruitment should be further evaluated, particularly in the WNND.

CCL11 (eotaxin-1) is a chemokine that selectively recruits eosinophils to sites of inflammation and binds to three receptors CCR2, CCR3, and CCR5. By interacting with IL-3, IL-5, and GM-CSF (granulocyte-macrophage colony-stimulating factor), CCL11 regulates the differentiation, migration, and biological activity of eosinophils. Elevated concentrations of CCL11 have been associated with psychiatric disorders and ROS/RNS-induced oxidative stress, which represents a factor in promoting major depressive disorder and phenotypic switching. However, the possible biological effect of this chemokine in the pathogenesis of neuroinvasive CNS infections, including TBE and WNND, remains unclear [50].

In this study, we did not observe significant differences in the expression of analyzed chemokines according to the clinical presentation (meningitis vs. meningoencephalitis) or disease outcome in WNND. However, our results regarding the association between selected chemokines and disease outcome are limited due to a small number of fatal outcomes observed in this study (4.8% of WNND patients, none in the TBE group), and this issue needs to be further evaluated in other studies.

Analysis of CSF chemokine and cytokine composition and a possible application of selected molecules as biomarkers of diagnosis, clinical severity, and treatment outcome in various inflammatory and noninflammatory neurological diseases need further evaluation. Studies in rapidly progressing CNS lymphoma (CNSL) have recently confirmed the excellent diagnostic utility of CXCL13 as well as CXCL9 as predictors of diagnosis, severity of the clinical presentation, and response to therapy. For example, by using logistic regression and a minimal-p-value approach, Masouris et al. (2021) [51] have shown that a cut-off value of 80 pg/mL of CXCL13 shows a 90.7% sensitivity and 90.1% specificity for the diagnosis of active CNSL, suggesting a possibility for real-world diagnostic use of this chemokine in CNSL. A recent retrospective cross-sectional study that included 1234 patients undergoing a lumbar puncture demonstrated the highest diagnostic performance of CSF CXCL13 concentration as an activity marker for acute neuroborreliosis (92.1% sensitivity, 96.5% specificity). However, the study also demonstrated that elevation of CXCL13 is not specific for neuroborreliosis but is also present in neurosyphilis, cryptococcal meningitis, and primary/secondary B-cell lymphoma, suggesting the need for careful interpretation of results in this context [52]. In addition, the potential diagnostic value of CXCL13 as a biomarker reflecting disease activity was recently evaluated in multiple sclerosis [53]. Data on the diagnostic value of CSF chemokines and cytokines in pediatric cohorts are limited. Recently, CCL2, CXCL8, CXCL10, CXCL13, and IL-6 in the CSF have been evaluated as biomarkers of CNS inflammation in a cohort of children with diverse (mostly non-infectious) inflammatory CNS diseases. Interestingly, CXCL13 was identified as a biomarker with the highest predictive utility for the general recognition of neuroinflammation in the particular cohort [54]. Recently, a systematic review and meta-analysis by Ma et al. (2023) [55] showed increased CSF concentrations of CXCL10 in patients with anti-N-methyl-D-aspartate receptor encephalitis (NMDAR-E) compared with patients presenting with non-inflammatory neurological disorders, suggesting its possible diagnostic value in this model as well.

The ability of an infected host to synthesize chemokines, cytokines, and other immunological mediators in response to viral infection may be influenced by age, genetics, and features of the immune system, which represents a limitation of studies in this scientific area. We identified one meta-analysis by Hoffman et al. (2019) [56] that reported gender-based differences in the frequency of clinical symptoms following WNV infection in a cohort of 115 blood donors from the USA (more symptoms reported by females). Furthermore, the authors also found gender-based differences in systemic chemokine responses with WNV-infected males in the post-IgM phase showing increased concentrations of CXCL10 and other chemokines (CCL2 and CCL11) compared to WNV-infected females as well as compared to uninfected males. In addition, a positive correlation was observed between CCL11 and age among WNV-infected males (but not among females). Of note, the differences in the gender-based cytokine profiles were not influenced by greater initial viral replication or innate host responses (no significant differences in plasma viremia and concentrations of interferon-α were found). These results may suggest that despite initial similarities in the response to WNV in males and females, sex differences in the immune responses observed as the infection progresses may impact the clinical outcome. Contrary to these findings, we failed to find a significant correlation between intrathecal concentrations of 12 selected chemokines and both age and sex in WNND and TBE groups in the acute stage of infection. However, based on data reported by Hoffman et al. (2019) [56] in asymptomatic blood donors, longitudinal monitoring of systemic chemokine concentrations of WNND and TBE patients during the post-recovery period might prove very interesting.

## 5. Conclusions

In conclusion, this study has shown, for the first time, similar patterns of CSF chemokine expression in WNND and TBE, particularly those regulating the recruitment of monocytes/macrophages (CCL2), neutrophils (CXCL1 and CXCL8), and leukocytes (CXCL10 and CXCL11) to sites of inflammation. The discovery of common CSF chemokine signatures in WNND and TBE possibly suggests common local immunopathogenic mechanisms in human neuroinvasive flavivirus infections that should be investigated in other disease models as well. In addition, we have shown that CSF concentrations of CCL11, CXCL5, and CXCL10 can be used as criteria for patient classification as WNND or TBE versus other neuroinvasive diseases. Evaluation of these chemokines as possible biomarkers of clinical severity of disease or mortality in neuroinvasive flavivirus infections seems warranted.

## Figures and Tables

**Table 1:** Demographic and clinical characteristics of patients with confirmed flavivirus infection.

Parameter		All Patients (N = 62)	Patients with TBEV (N = 31)	Patients with WNV (N = 31)
Demographics	Male sex	38 (61%)	21 (68%)	17 (55%)
	Age (median, range; years)	58 (12–88)	46 (12–74)	63 (13–88)
Clinical characteristics and outcome	Meningitis	31 (50%)	15 (48%)	16 (52%)
	Meningoencephalitis	23 (37%)	12 (39%)	11 (36%)
	Febrile headache	4 (7%)	3 (10%)	1 (3%)
	Myelitis	2 (3%)	1 (3%)	1 (3%)
	Poliradiculoneuritis	2 (3%)	0 (0%)	2 (7%)
	Fatal outcome	3 (5%)	0 (0%)	3 (10%)

TBEV = tick-borne encephalitis virus, WNV = West Nile virus.

**Table 2:** Binary logistic regression model classifying TBEV + WNV patients and patients with non-determined NID etiology.

Chemokine	Coefficient	Standard Error	*p*-Value
CCL11	0.135	0.043	<0.001
CXCL5	−0.144	0.034	<0.001
CXCL10	−0.043	0.015	0.004

## Data Availability

Data are contained within the article.
